# Editorial: A New Phase for *JNCI Cancer Spectrum*

**DOI:** 10.1093/jncics/pkab006

**Published:** 2021-02-19

**Authors:** Ronald C Chen

**Affiliations:** Department of Radiation Oncology, University of Kansas Medical Center, Kansas City, KS, USA

Welcome to the fifth year of *JNCI Cancer Spectrum*! Launched in 2017 by the nonprofit organization Oxford University Press, the Journal is fully open access with a reasonable publication fee for authors, consistent with calls from international organizations for free and wide dissemination of research findings not only to researchers but also to patients. The editors of the Journal are committed to provide rapid and rigorous peer review of submitted manuscripts and publish accepted papers online in a timely fashion so important cancer research can quickly start to make an impact.

## Content Focus of the Journal

Along with our sister journal, *Journal of the National Cancer Institute* (*JNCI*), *JNCI Cancer Spectrum* seeks to publish high-quality original research as well as timely reviews and commentaries. The Journal publishes a broad spectrum of cancer research that has direct relevance to cancer patient care and/or health policy, ranging from population health and epidemiology to clinical (both prospective and retrospective) and translational research. The editors encourage direct manuscript submissions to the Journal, which also provides a seamless transfer process for manuscripts that are deemed of high quality but not of sufficient publication priority for *JNCI*. In addition, we encourage submission of well-conducted studies that either report a novel finding or confirm or validate previously reported findings as well as clinical trials with either “positive” or “negative” results.

## Journal’s Growth

**Figure 1.**
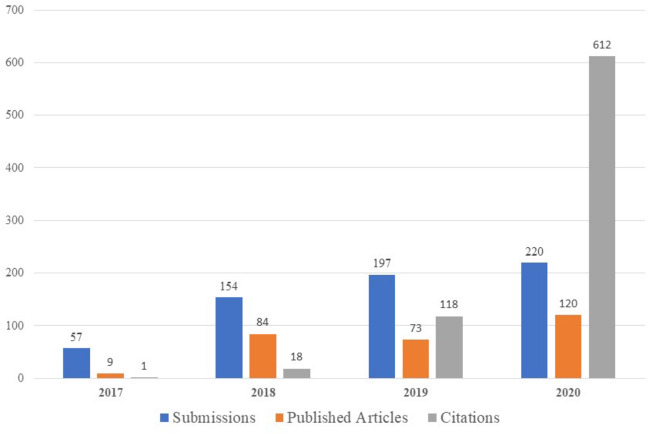
Submissions, Published Articles, and Citations for JNCICS from 2017-2020.

Under Dr Pamela Goodwin’s leadership as the inaugural Editor-in-Chief, the Journal published its first issue in September 2017. It is currently published 6 times per year. The Journal has experienced tremendous growth in the past 4 years by all metrics, including submissions, published articles, and citations (Figure 1). In 2020, the Journal successfully gained indexing on Scopus and PubMed Central, which means that all of the Journal publications are now easily searchable on these platforms. The Journal also has been selected for the Emerging Sources Citation Index, which places it in the Web of Science Core Collection. With these preliminary steps, the Journal will hopefully gain its first Impact Factor in the near future.

As Dr Goodwin completed her term as Editor-in-Chief at the end of 2020, the Journal is primed for continued and increasing success. I have been working with Pam since the Journal’s inception, initially as an Associate Editor and then as Deputy Editor. I want to thank Oxford University Press for giving me the honor and opportunity to lead this journal in its next phase; Pam for her strong leadership over the past 4 years; and the hard work of the Journal’s esteemed and international group of staff, editors, and reviewers—who together have built this journal from its beginning. Most of all, I want to thank you, the authors—because you are the foundation of the Journal—and the readers, the Journal’s *raison d'être*.

I invite you to submit your manuscript to the Journal and welcome your feedback and input for the Journal as it continues to grow in its exciting next phase.

